# Dysbiosis and *Staphylococcus* species over representation in the exit site skin microbiota of hemodialysis patients carrying tunneled cuffed central venous catheter

**DOI:** 10.1080/0886022X.2024.2363417

**Published:** 2024-06-24

**Authors:** Bai-li Xiao, Xue-Qing Hu, Ming Li

**Affiliations:** a Department of Blood purification, The Affiliated Yantai Yuhuangding Hospital of Qingdao University, Yantai, Shandong, China; b Department of Blood purification, Qingdao Municipal Hospital, Qingdao, Shandong, China; c Department of Gastroenterology, Qilu Hospital, Cheeloo College of Medicine, Shandong University, Jinan, Shandong, China; d Laboratory of Translational Gastroenterology, Qilu Hospital, Cheeloo College of Medicine, Shandong University, Jinan, Shandong, China; e Robot Engineering Laboratory for Precise Diagnosis and Therapy of GI Tumor, Qilu Hospital, Cheeloo College of Medicine, Shandong University, Jinan, Shandong, China

**Keywords:** Hemodialysis, skin microbiota, metagenome, tunneled cuffed catheters, dysbiosis

## Abstract

**Objectives:**

Hemodialysis patients with end-stage renal disease (ESRD) are susceptible to infections and dysbiosis. Catheter-related infections are typically caused by opportunistic skin pathogens. This study aims to compare the skin microbiota changes around the exit site of tunneled cuffed catheters (peri-catheter group) and the contralateral site (control group).

**Methods:**

ESRD patients on hemodialysis were recruited. The skin microbiota were collected with moist skin swabs and analyzed using high-throughput sequencing of the 16S rDNA V3–V4 region. After denoising, de-replication, and removal of chimeras, the reads were assigned to zero-radius operational taxonomic units (ZOTU).

**Results:**

We found significantly reduced alpha diversity in the peri-catheter group compared to the control group, as indicated by the Shannon, Jost, and equitability indexes, but not by the Chao1 or richness indexes. Beta diversity analysis revealed significant deviation of the peri-catheter microbiota from its corresponding control group. There was an overrepresentation of Firmicutes and an underrepresentation of Actinobacteria, Proteobacteria, and Acidobacteria at the phylum level in the peri-catheter group. The most abundant ZOTU (*Staphylococcus* spp.) drastically increased, while *Cutibacterium*, a commensal bacterium, decreased in the peri-catheter group. Network analysis revealed that the skin microbiota demonstrated covariance with both local and biochemical factors.

**Conclusions:**

In conclusion, there was significant skin microbiota dysbiosis at the exit sites compared to the control sites in ESRD dialysis patients. Managing skin dysbiosis represents a promising target in the prevention of catheter-related bacterial infections.

## Introduction

More than 100 trillion microbes inhabit various niches of our body, including the oral cavity, skin, and gastrointestinal tract [1]. Humans coexist with their microbiota, communities of bacteria, fungi, and viruses [2]. The microbiome significantly influences the occurrence and development of diseases [3,4]. The impact of gut microbiota on inflammatory bowel disease (IBD) and other digestive diseases has been extensively studied [5,6]. Recently, the importance of the skin microbiota has gained attention. The skin microbiota closely interacts with the host and communicates with various cutaneous cells, regulating wound healing and barrier restoration [7]. Skin commensals are now considered essential for regulating the cutaneous immune system [7]. Chronic kidney disease patients experience dysbiosis in both the gut [8] and the skin [9] microbiota, leading to metabolic disturbances and skin symptoms such as itching. Furthermore, chronic wounds caused by subcutaneous catheter tunnels disrupt the barrier between the skin microbiota and the internal environment in end-stage renal disease (ESRD) patients on maintenance hemodialysis. Thus, the skin microbiota around the exit site is extremely vulnerable and requires special attention.

Catheter-related infection is a complication that significantly increases hospitalization rates and mortality in hemodialysis patients with central venous catheters [10]. Several risk factors contribute to catheter-related infections, including long-term hospitalization, prolonged catheter retention, improper dressing, local blood residue, an inadequate sterile barrier during catheter placement, poor catheter blood flow during dialysis, and repeated adjustments of catheter position. Despite adherence to strict aseptic techniques, catheter-related bloodstream infections continue to occur at unacceptable rates [10]. This suggests that there may be additional factors yet to be addressed. Dysbiosis in barrier organs is often associated with infection. Changes in vaginal microbiota characteristics, resulting in the loss of protective Lactobacillus spp., increase the risk of urinary tract infection [11]. Similarly, altered gut bacteria composition is associated with the pathogenesis of many inflammatory diseases and infections [12]. However, the role of peri-catheter skin microbiota in catheter-related infections has not yet been studied extensively.

In this study, we posed the question of whether dysbiosis exists in the peri-catheter skin microbiota of ESRD patients with tunneled-cuffed catheters for maintenance hemodialysis and whether peri-catheter skin dysbiosis is associated with adverse clinical outcomes. To address this question, we established a preliminary cohort and profiled their skin microbiota through high-throughput pyrosequencing. We believed these results would reveal previously unnoticed clinical conditions in ESRD patients.

## Materials and methods

### Study cohort and ethics statement

ESRD patients with tunneled cuffed catheters for maintenance hemodialysis were recruited from the Blood Purification Department of the Affiliated Yantai Yuhuangding Hospital of Qingdao University between August and September 2022. The inclusion criteria were: (1) patients on maintenance hemodialysis through long-term central venous catheters, (2) willingness to provide skin microbiota samples, (3) fully informed participants who signed a written informed consent form, and (4) subjects older than 18 years. The exclusion criteria were: (1) systemic use of antibiotics within 30 days, (2) systemic use of immunosuppressants and biologic agents, (3) external drug use at the sampling site, (4) pregnancy, puerperium, or lactation, (5) presence of other unstable serious diseases, and (6) failure to collect skin microbiota samples at the peri-catheter region or control sites. This study was approved by the Medical Ethics Committee of the Affiliated Yantai Yuhuangding Hospital of Qingdao University (2022-296), and all subjects signed informed consent forms.

### Data and sample collection

For each participant, demographic data, primary renal disease, complications, and dialysis age were recorded at enrollment. Skin conditions and microbiota were studied in two regions: (1) the peri-catheter region, a 2 × 2 cm area around the exit site of the tunneled cuffed catheter, and (2) the control region, a 2 × 2 cm area contralateral to the corresponding peri-catheter region. To collect the skin microbiota, sterile cotton swabs were soaked in sterile saline and rubbed vigorously on the pre-specified skin sites for 20 s. The swabs were then immediately placed in DNA preservation tubes, which maintain the DNA samples at room temperature for one week (Cat# GDS-01A TinyGene, Shanghai, China). After the initial data and sample collection, all participants were followed up for 15 months. Any deaths or infections were recorded.

### DNA extraction and amplification

After each sample was collected, the preservation tube was packed with ice and transported to Novogene® (Beijing, China) within 48 h. The samples were stored at −80 °C until microbial DNA was extracted. Total genome DNA from samples was extracted using the CTAB method. DNA samples were verified by micro ultraviolet spectrophotometer and 1% agarose gel electrophoresis. The V3–V4 region of the 16S rRNA gene was amplified using specific primers (341 F, CCTAYGGGRBGCASCAG and 806 R, 5′-GGACTACNNGGGTATCTAAT-3′. All PCR reactions were carried out with 15 μL of Phusion® High-Fidelity PCR Master Mix (New England Biolabs); 0.2 μM of forward and reverse primers, and about 10 ng of template DNA. Thermal cycling consisted of initial denaturation at 98 °C for 1 min, followed by 30 cycles of denaturation at 98 °C for 10 s, annealing at 50 °C for 30 s, and elongation at 72 °C for 30 s. The PCR products were mixed with the same volume of IX loading buffer and underwent electrophoresis in 2% agarose gel. The PCR products were mixed in equidensity and then purified with the Qiagen Gel Extraction Kit (Qiagen, Germany).

After collection, each sample was packed with ice and transported to Novogene® (Beijing, China) within 48h. The samples were stored at −80 °C until microbial DNA extraction. Total genome DNA from samples was extracted using the CTAB method. DNA samples were verified by micro ultraviolet spectrophotometer and 1% agarose gel electrophoresis. The V3–V4 region of the 16S rRNA gene was amplified using specific primers (341 F, CCTAYGGGRBGCASCAG, and 806 R, 5′-GGACTACNNGGGTATCTAAT-3′). All PCR reactions were carried out with 15 μL of Phusion® High-Fidelity PCR Master Mix (New England Biolabs), 0.2 μM of forward and reverse primers, and about 10 ng template DNA. Thermal cycling consisted of initial denaturation at 98 °C for 1 min, followed by 30 cycles of denaturation at 98 °C for 10 s, annealing at 50 °C for 30 s, and elongation at 72 °C for 30 s, concluding with a resting period at 72 °C for 5 min. The PCR products were mixed with the same volume of 1X loading buffer and underwent electrophoresis in 2% agarose gel. The PCR products were then mixed in equal density and purified with the Qiagen Gel Extraction Kit (Qiagen, Germany).

### 16S rRNA gene sequencing

Sequencing libraries were generated using the TruSeq® DNA PCR-Free Sample Preparation Kit (Illumina, USA) following the manufacturer’s recommendations, with index codes added. The library quality was assessed using the Qubit@2.0 Fluorometer (Thermo Scientific) and the Agilent Bioanalyzer 2100 system. Finally, the library was sequenced on an Illumina NovaSeq platform, generating 250 bp paired-end reads.

### Sequence processing and microbiota classification

Paired-end reads were assigned to samples based on their unique barcode and truncated by cutting off the barcode and primer sequence. Paired-end reads were merged using FLASH (VI.2.7, http://ccb.jhu.edu/software/FLASH/) [13] with a minimum required overlap length of 10 bp. The splicing sequences were called raw tags. Quality filtering on the raw tags was performed to obtain high-quality clean tags according to FASTP [14]. The minimal quality value for a base was set at 19, as indicated by the parameter -q 19. Valid sequences were downloaded for microbiota profiling. All valid sequencing data were processed using Usearch (version 11.0.667) software [15]. The valid sequences were de-replicated using the fastx_uniques algorithm of Usearch. The unique reads were denoised, and chimeras were removed using the unoise3 command to produce zero-radius operational taxonomy units (ZOTUs). The representative sequence of each ZOTU was aligned to the RDP (version 18) using the sintax algorithm with a sintax_cutoff of 0.8. The ZOTU abundance figure was merged at the genus and phylum levels using the sintax_summary algorithm with parameters -rank g and -rank p, respectively. Alpha and beta diversities were calculated using the Usearch -alpha_div, -cluster_agg, and -beta_div algorithms. Non-metric multidimensional scaling (NMDS) with adonis analysis was performed with the metaMDS, adonis, or anosim functions of the vegan package v2.5 in R. LEfSe (linear discriminant analysis [LDA] coupled with effect size measurements) analysis was conducted to determine the biomarkers in each group. The starting formatted data file for LEfSe analysis was made from the Usearch-produced phylum, class, order, family, and genus level summary files using handcrafted code in R. By running the LEfSe script from GitHub (https://github.com/SegataLab/lefse/tree/master/lefse) in python3, the LDA score of biomarkers was calculated and plotted. A stricter all-against-all strategy was adopted for the multi-class comparison. Biomarkers with an LDA score > 3.0 were filtered and plotted.

### Statistical analysis

Data are reported as the median ± SEM. Statistical analyses were conducted using R software (version 4.2.1). Differences between paired groups were evaluated using the paired Wilcoxon test, with *p* values <.05 considered significant. The co-occurrence network of the top 50 most abundant ZOTUs and environmental factors was visualized to explore host-microbiota interactions. The Spearman correlation between ZOTU abundances was calculated using the cor.test function in R. A strict *p* value threshold (*p* < .01) was applied to filter strong correlations, and the filtered *p* values were adjusted using the fdr method in R. Significant correlations with adjusted *p* values were exported to Cytoscape V.3.2.1. Each node represents a ZOTU in the microbiota network, with its diameter proportional to the square root of the mean abundance of all samples. Solid and dashed edges represent positive and negative correlations, respectively, and nodes were positioned according to the weighted perfuse force-directed layout.

## Results

### Demographic and clinical characteristics of the study cohort

Seventeen recipients were enrolled in this study, with seven excluded due to failure to obtain sequencing data from the skin microbiota at the peri-catheter region or control sites. All participants were of Asian ethnicity and resided in Shandong Province, China. The demographic data, dialysis history, and follow-up information are listed in Table 1. All 10 patients had suture fixation for the catheter, with nine experiencing skin redness around the exit site. One patient’s catheter exit site was not covered with dressing for an extended period, while the others were consistently covered with surgical dressings.

**Table 1.** Baseline and follow up data of enrolled patients.

Num	Baseline indices									Follow up data				
	Primary disease	DM	Gender	Age (year)	Height (cm)	BW (kg)	Dialysis duration (month)	catheter duration (month)	Time to last hospitalization (month)	Survival (month)	death reason	Viral infection	Blood culture	Sputum culture
1	DKD	+	Female	70	158	62.4	42.1	38.1	39.8	>15		Covid-19		
2	DKD	+	Male	69	165	69.9	29.2	29.2	6.1	>15		Covid-19	*Corynebacterium afermentans*	*Acinetobacter baumannii,* *Pseudomonas aeruginosa*
3	HN	−	Male	80	168	80.3	28.1	25.1	25.6	3	Cardiogenic shock			
4	CGN	−	Male	84	176	88	112.0	9.4	111.0	13	Appendicitis and infectious shock			
5	ADPKD	−	Male	57	180	72.3	96.0	9.0	7.6	>15		Covid-19		
6	HN	−	Female	89	150	71	15.8	8.9	14.2	6	Respiratory failure	Covid-19		
7	DKD	+	Male	74	173	84.2	28.5	28.5	28.2	<1	Sudden death			
8	DKD	+	Male	70	170	69.7	18.6	17.0	3.4	5	Sudden death	Covid-19		
9	MDS,NS	−	Male	66	175	72.8	19.0	16.0	3.0	>15				
10	DKD	+	Male	79	168	67.4	16.1	16.1	15.0	12	Sepsis and respiratory failure		*Staphylococcus epidermidis*	*Acinetobacter pittii,* *Klebsielia oxytoca,* *Acinetobacter baumannii*

*Note:* DM: diabetes mellitus; DKD: diabetic kidney disease; HN: hypertensive nephropathy; CGN: Crescentic glomerulonephritis; ADPKD: autosomal dominant polycystic kidney disease; MDS: myelodysplastic syndrome.

During the follow-up period, six patients died from causes including cardiogenic shock, appendicitis, septic shock, respiratory failure, and sudden death. Five of these patients were positive for Covid-19, and two had documented bacterial infections (Table 1). These findings align with previous reports that dialysis patients are vulnerable to infections.

### Alpha diversity reduction in the skin microbiota

**Figure 1.**
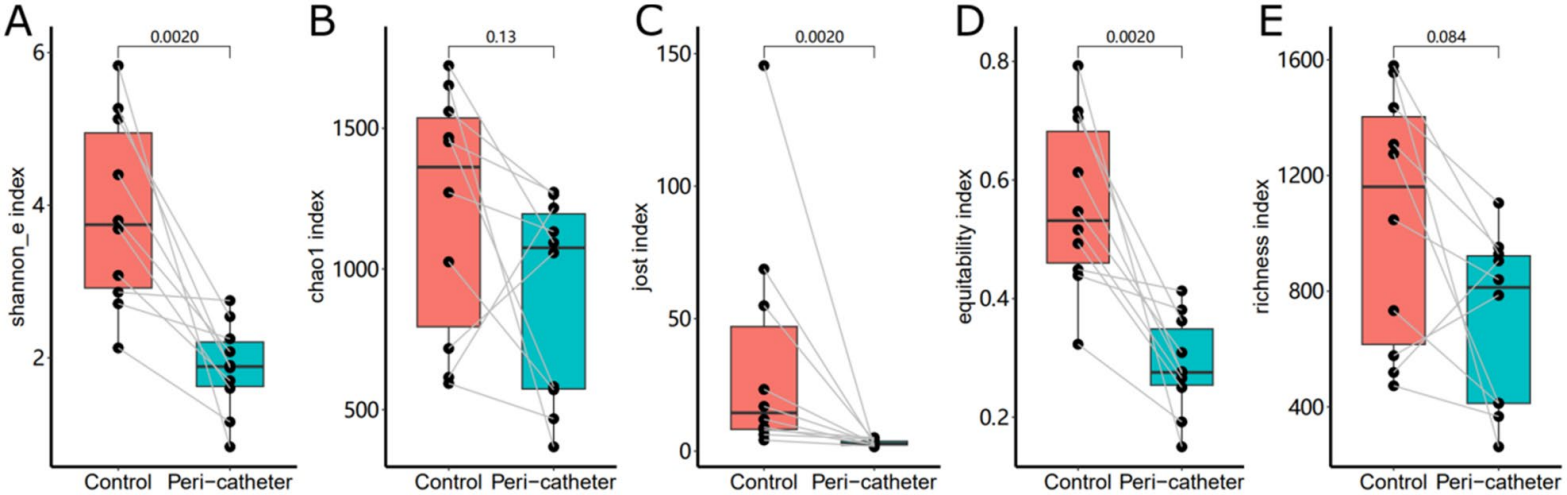
Alpha diversity of the skin microbiota. The Shannon_e (A), Chao1 (B), Jost (C), equitability (D), and richness (E) indexes were compared between the peri-catheter and the contralateral control sites. Paired Wilcoxon tests were conducted, and the *p* values are labeled in each plot.

Sequencing of the 16S rRNA gene yielded 1,255,467 valid reads from 20 successfully sequenced samples, generating 346,251 unique reads after de-replication and 2741 ZOTUs after chimera removal. Comparison of alpha diversity (Figure 1) between the control sites and peri-catheter skin microbiota revealed significantly lower Shannon_e index (median 1.885 vs. 3.745, *p* = .0020) and Jost index (median 2.7 vs. 14.45, *p* = .0020) in the peri-catheter group. The Chao1 index was insignificantly lower in the peri-catheter skin than in the controls (median 1075 vs. 1362, *p* = .1309). Similarly, microbiota equitability (median 0.2755 vs. 0.5315, *p* = .0020), but not richness (median 813 vs. 1161, *p* = .0840), was significantly reduced at the catheter exit site compared to the controls. There was no significant correlation between catheter duration and these alpha diversity indices (all *p* > .2 by Spearman test). These findings indicate decreased alpha diversity and predominantly low equitability in the peri-catheter skin microbiota of hemodialysis patients.

### Beta diversity disturbance of the peri-catheter skin microbiota

**Figure 2.**
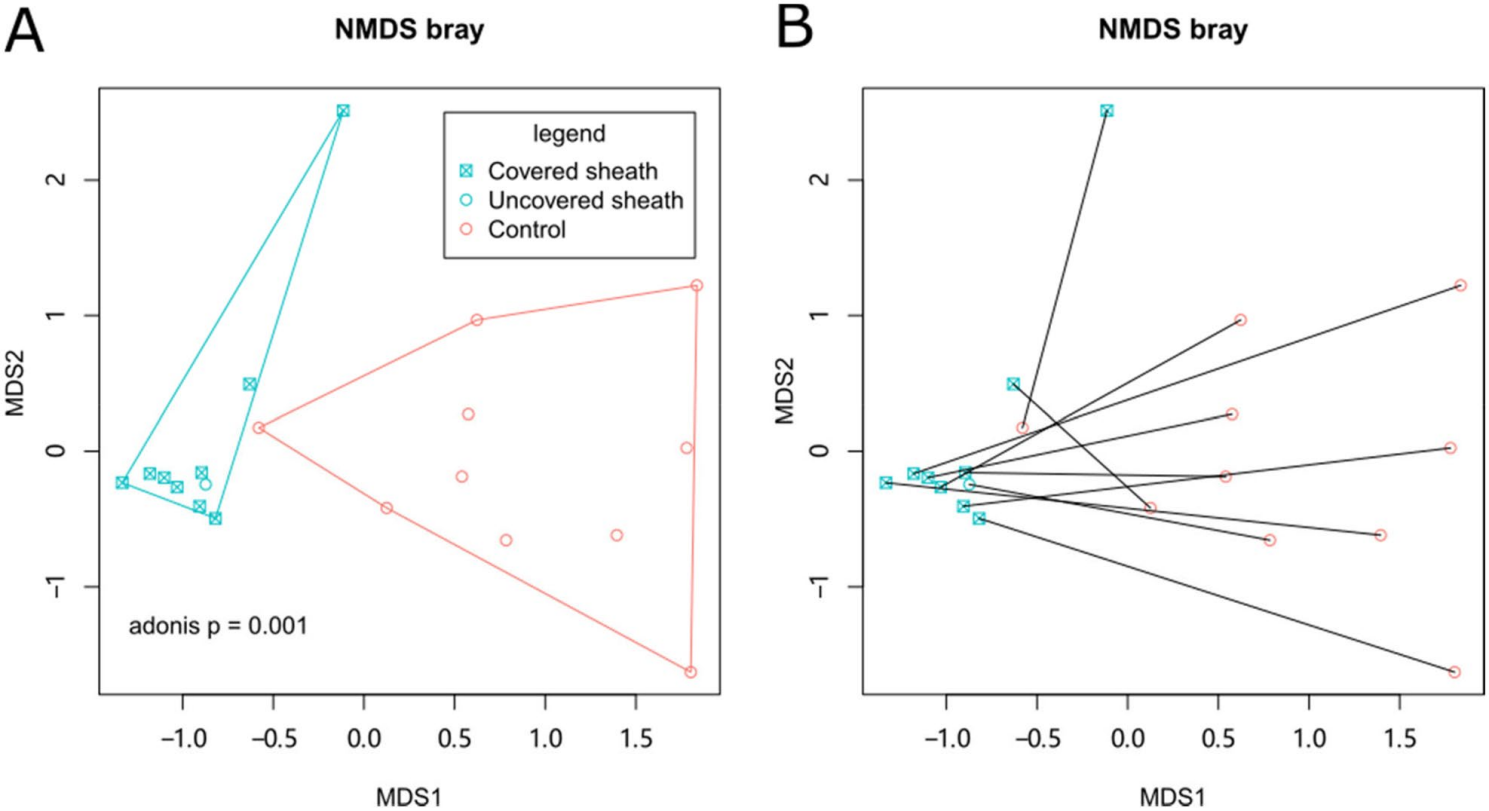
The beta diversity of skin microbiota. NMDS analysis was performed based on Bray distance. The 2-D coordinates were plotted with polygons indicating the groups (A) or with lines connecting paired samples from the same patients (B).

Differences in the beta diversity of skin microbiota between groups were assessed by filtering the top 20 OTUs and performing a Bray distance-based NMDS analysis. This analysis revealed that the peri-catheter skin microbiota differed from the controls, as represented by two non-overlapping convex hulls in Figure 2(A). This finding was confirmed by the Adonis test (*p* = .001). The PERMDISP2 procedure and ANOVA test revealed that the control skin microbiota was more dispersed than the peri-catheter microbiota (*p* = .001462), consistently shown by the larger convex hull of the control group in Figure 2(A). The presence or absence of surgical dressing covering the peri-catheter skin did not affect the microbiota, as indicated by NMDS analysis (Adonis *p* = 1, Figure 2(A)). Nor did the catheter duration affect the skin microbiota (*p* = .555 by single-factor Adonis test). Re-plotting the NMDS coordinates connected for corresponding peri-catheter and control pairs showed that these paired coordinates did not cluster closely (Figure 2(B)). An Adonis model including six additional biochemical factors (albumin, creatinine, BUN, CRP, β2-microglobulin, and pre-albumin) revealed that none significantly contributed to beta-diversity. These data collectively suggest that the skin microbiota at the peri-catheter site is similar yet distinct from normal skin, and these differences are primarily influenced by local factors (with or without the catheter) rather than systemic factors or surgical dressing coverage.

### Taxonomical changes of the skin microbiome

**Figure 3.**
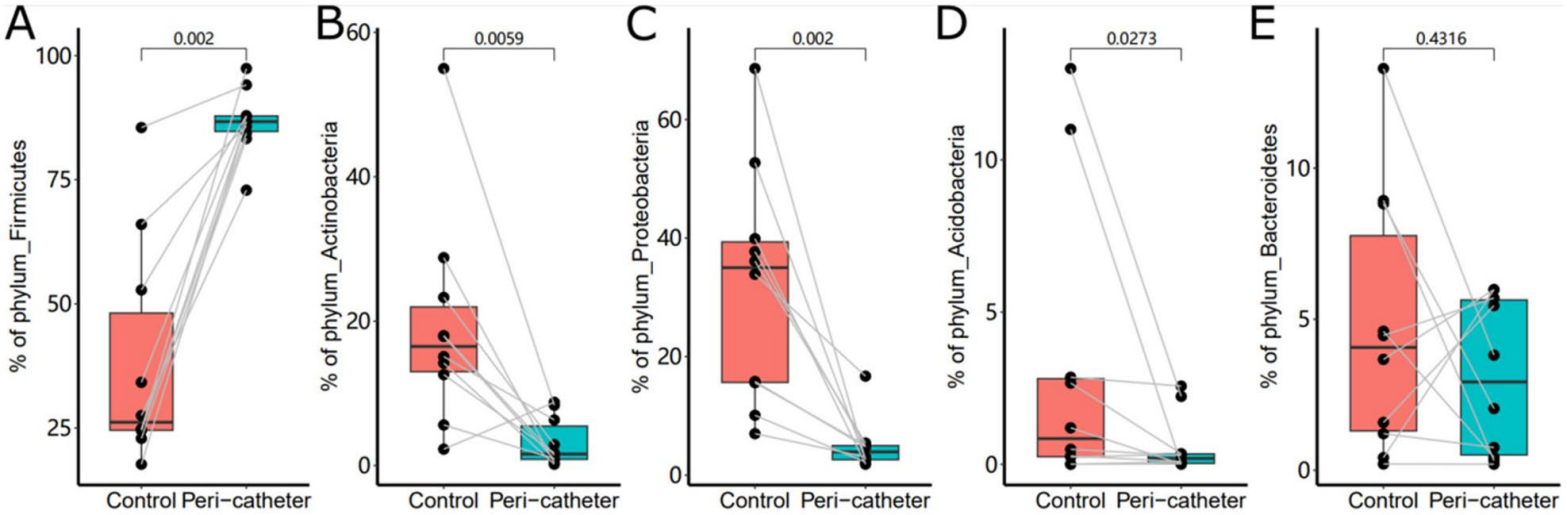
The abundance of the major phyla in the skin microbiota. The phyla Firmicutes (A), Actinobacteria (B), Proteobacteria (C), Acidobacteria (D), and Bacteroidetes (E) were compared between the peri-catheter and the contralateral control sites. Paired Wilcoxon tests were conducted, and the *p* values are labeled in each plot.

Taxonomical profiling of the skin microbiota started at the phylum level. The five most abundant phyla, Firmicutes, Actinobacteria, Proteobacteria, Bacteroidetes, and Acidobacteria, accounted for 91.88% to 99.86% of the microbiota in the studied samples. We found a drastically increased abundance of Firmicutes in the peri-catheter group compared to the controls (86.7% vs. 26.2%, *p* = .0020, Figure 3(A)). Meanwhile, we observed significantly decreased abundances of Actinobacteria (1.61% vs. 16.5%, *p* = .0059, Figure 3(B)), Proteobacteria (3.93% vs. 35.0%, *p* = .0020, Figure 3(C)), and Acidobacteria (0.194% vs. 0.846%, *p* = .0273, Figure 3(D)) in the peri-catheter group. There was no significant difference in Bacteroidetes (*p* = .4316, Figure 3(E)). These data indicate a shift to Firmicutes-dominated microbiota in the peri-catheter skin.

**Figure 4.**
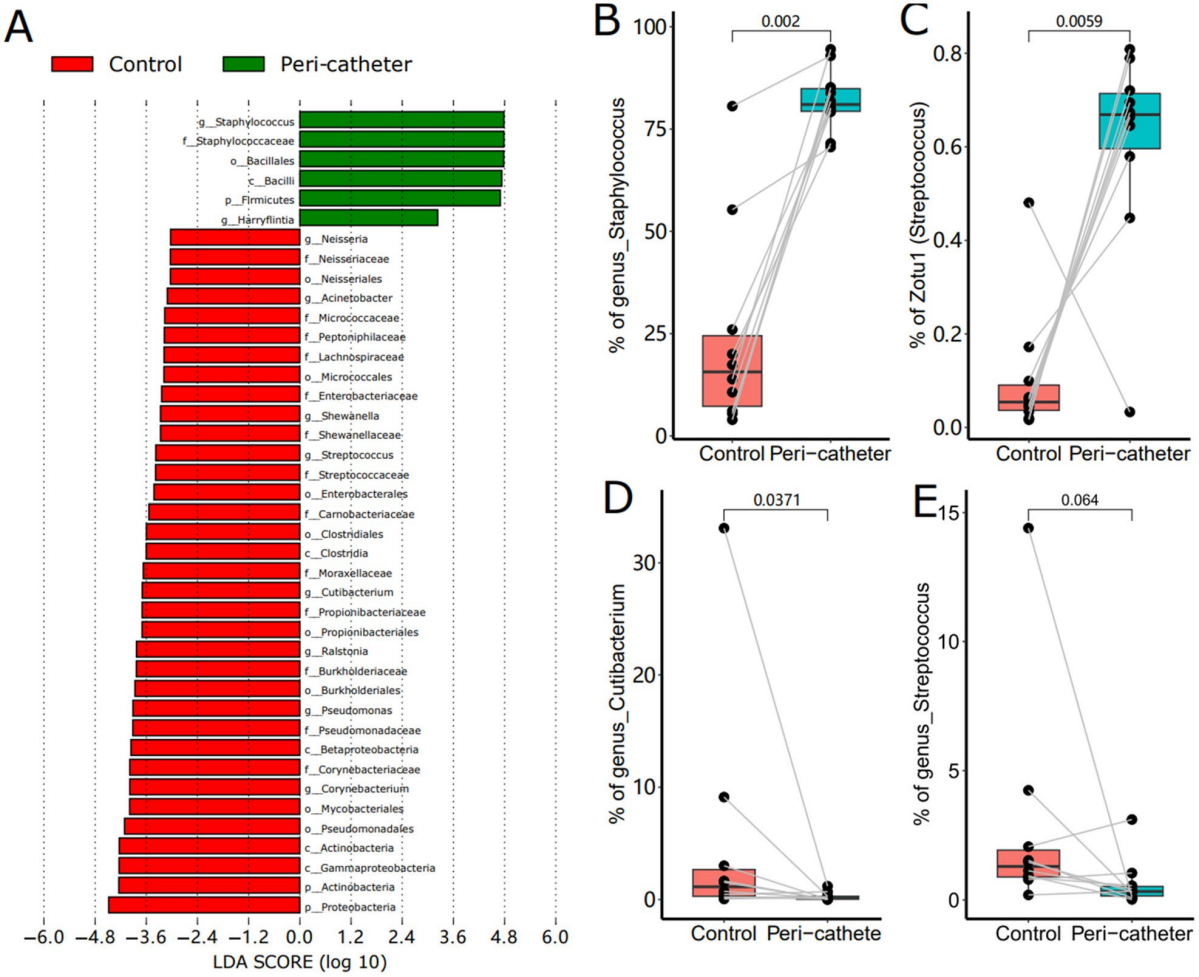
The LEfSe Analysis of the skin microbiota. Biomarkers with LDA score absolute values greater than 3 were plotted as bars (a). The abundance of Staphylococcus (B), Zotu1 (C), Cutibacterium (D), and Streptococcus (E) were compared between the peri-catheter and the contralateral control sites. Paired Wilcoxon tests were conducted, and the *p* values are labeled in each plot (B ∼ E).

To fully understand the microbiota changes at all taxonomical levels, we performed LEfSe analysis and filtered the differential taxonomy with LDA score > 3.0 (Figure 4(A)). In the peri-catheter group, we found significantly increased genera of *Staphylococcus* and *Harryflintia*, in the family of Staphylococcaceae and the order of Bacillales. The control group had more abundant genera of *Corynebacterium, Pseudomonas, Ralstonia, Cutibacterium, Streptococcus, and Neisseria* (Figure 4(A)). In the peri-catheter group, the abundance of *Staphylococcus* increased to 81.0% from 15.7% in the controls (*p* = .002, Figure 4(B)). The most abundant ZOTU (ZOTU1) was annotated as *Staphylococcus* sp., significantly higher in the peri-catheter group than in the controls (66.9% vs. 5.43%, *p* = .0059, Figure 4(C)). The genus abundance of *Cutibacterium*, a commensal bacterium, decreased in the peri-catheter group (0.128% vs. 1.14%, *p* = .0371, Figure 4(D)). The abundance of another commensal genus, Streptococcus, tended to decrease in the lesion samples compared to the control samples (0.327% vs. 1.30%, *p* = .0645, Figure 4(E)). These data suggest that *Staphylococcus* dominates among skin microbiota at the peri-catheter region.

### Network analysis between the microbiota and skin characteristics

**Figure 5.**
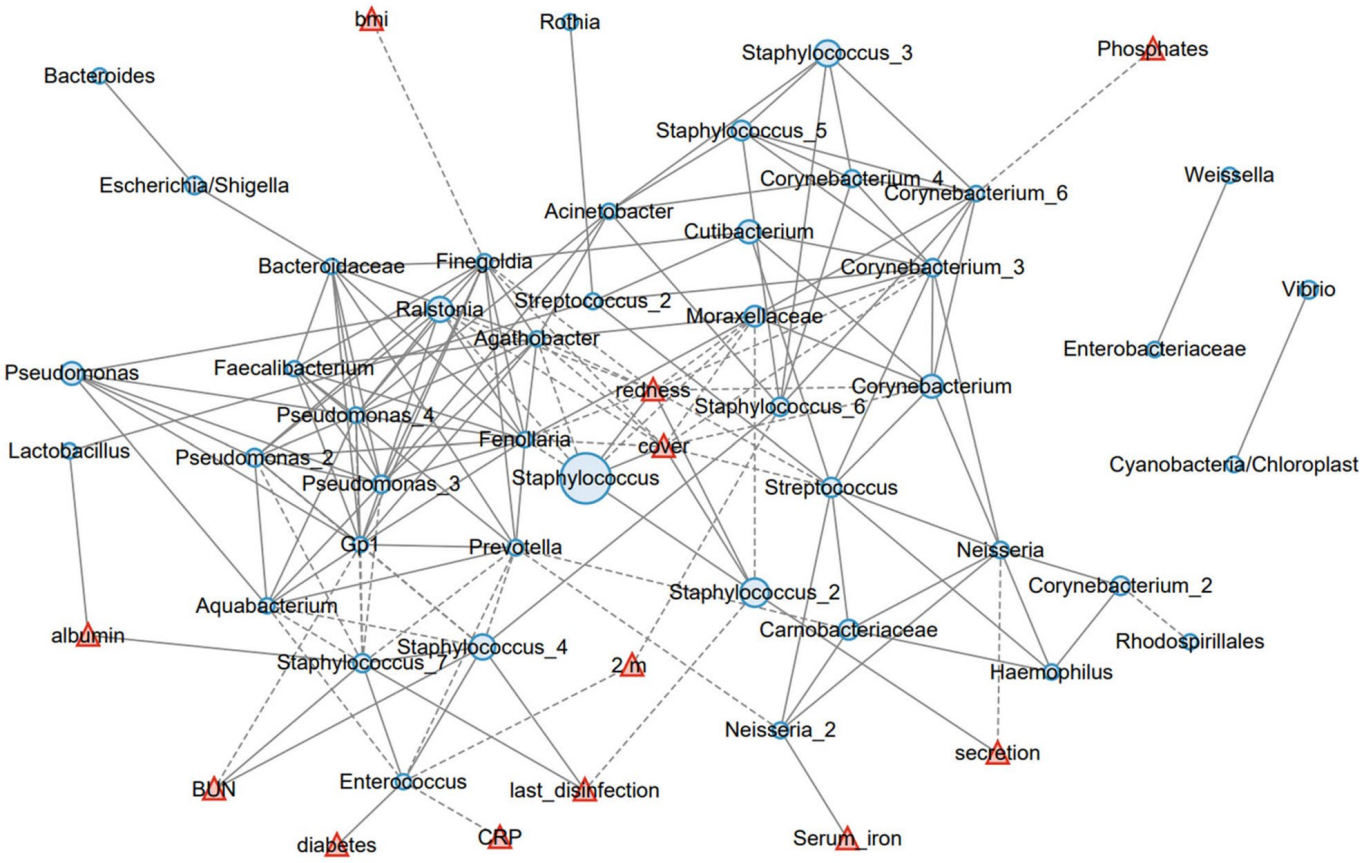
The network analysis involving the skin microbiota and environmental factors. The 50 most abundant ZOTUs are represented in light blue circles, with their diameters proportional to the square roots of the mean abundance in all involved samples. The environmental factors are plotted in red triangles. Solid lines represent positive correlations, and dashed lines represent negative correlations.

To comprehensively understand the interaction between the skin microbiota and systemic or local factors, we conducted a network analysis involving these factors. Within the microbiota, the most abundant *Staphylococcus* ZOTU negatively correlated with *Feenollaria, Ralstonia, Finegoldia*, and a Moraxellaceae ZOTU (Figure 5). It also positively correlated with another *Staphylococcus* ZOTU (designated as *Staphylococcus*_2). Four demographic factors, including age, BMI, sex, diabetes, and dialysis age; 10 local environmental factors, including the shower interval, last shower time, suture, cover, secretion, granulation, itchiness, last disinfection, redness, and catheter service time; and fifteen systemic biochemical factors, including transferrin saturation, RBC, hemoglobin, Hct, serum iron, transferrin, calcium, phosphates, PTH, albumin, pre-albumin, Cr, BUN, CRP, and β2m, were tested for their correlation with the skin microbiota components in the network analysis. We found that skin redness and coverage by medical dressings positively correlated with *Staphylococcus* and *Staphylococcus*_2. Local fluid secretion positively correlated with *Staphylococcus*_2 but negatively with Neisseria. The days since last disinfection positively correlated with two *Staphylococcus* ZOTUs (designated as *Staphylococcus*_7 and *Staphylococcus*_4, Figure 5). These results suggest that *Staphylococcus* is mainly affected by local environmental factors, such as inflammation and the use of disinfectants.

The systemic biochemical factors were also related with the skin microbiota. Albumin positively corelated with Lactobacillus and *Staphylococcus*_7. Serum BUN positively correlated with *Staphylococcus*_4 and *Staphylococcus*_7. Beta-2 microglobulin negatively correlated with *Enterococcus* and Staphylococcus_6. *Enterococcus* also negatively correlated with C-reactive protein (CRP). Serum iron positively correlated with a *Neisseria* ZOTU (*Neisseria*_2), and serum phosphates negatively correlated with a *Cornebacterium* ZOTU *(Cornebacterium*_6). No other studied factors were determined to be related with the skin microbiota. Collectively, these data showed that primarily environmental factors are involved in the imbalance between *Staphylococcus* and other cutaneous commensal bacteria.

## Discussion

In this study, we compared the skin microbiota around the exit site of the tunneled-cuffed catheter and the contralateral site in patients on maintenance hemodialysis. We found (1) significantly reduced alpha diversity, (2) a shift from Proteobacteria-dominated to Firmicutes-dominated microbiota, (3) over-representation of *Staphylococcus* species, and (4) co-variance between skin microbiota and local and biochemical factors. To our knowledge, this is the first study focusing on the skin microbiota around catheters. Understanding the skin microbiota and its dysbiosis is crucial to prevent or reduce skin-derived opportunistic bacterial infections in catheter-bearing patients.

ESRD patients on hemodialysis are vulnerable and require special attention to their dysbiosis and infection risks. Multiple factors may contribute to dysbiosis in this population. Immune system dysfunction is common among ESRD patients. A study by Zhao et al. documented increased Escherichia, Shigella, Desulfovibrio, and Streptococcus in the gut microbiota of CKD patients [16]. One of the uremic toxins, indoxyl 3-sulfate (I3S), accumulates in blood and tissues and suppresses Th2 immunity *via* the aryl hydrocarbon receptor (AhR) [17]. Previous surveys using 16S rRNA gene sequencing revealed that the chronic kidney disease (CKD) patients’ skin microbiota was significantly different from that of healthy controls’ [18]. Specifically, there was a depletion of bacterial diversity in the CKD group [18]. In our study, we used the contralateral site as an internal control for the peri-catheter skin microbiota. Our results show that the skin microbiota of control sites has a median Shannon value of 3.745, which is comparable to that of the CKD patients in Tian’s study (roughly 4.8 to 5.2 as interpreted from their figure) [18]. In our results, Firmicutes and Proteobacteria dominate the control skin microbiota, consistent with previous findings [18]. We found a further depletion of alpha diversity as indicated by the Shannon index. Thus, we may infer that the dysbiosis at the peri-catheter skin is caused by both systemic and local factors.

The skin microbiota fundamentally contributes to the skin barrier. The term microbiota represents the assemblage of microorganisms present in a specific environment, while the term microbiome refers to the entire habitat, including the microorganisms (bacteria, archaea, lower and higher eurkaryotes, and viruses), their genomes (i.e., genes), and the surrounding environmental conditions [19,20]. Human skin is an amazing structure composed of a stratified, cornified epithelium of keratinocytes, further fortified by chemical and immunological features that enhance the barrier [2]. The skin microbiota itself also forms a barrier against foreign and pathogenic microbes. The normal microbiota competes for the limited nutrient resources on skin, and some species, such as *Staphylococcus hominis*, produce antimicrobial peptides with potent inhibitory activity against the major skin pathogen *Staphylococcus aureus* [21]. The skin microbiota also regulates skin barrier function and repair *via* signaling through the aryl hydrocarbon receptor [22]. The microbes associated with the skin epithelium also cross-talk with innate immune cells through their metabolites. For example, the commensal bacterium *Cutibacterium acnes* ferments triglycerides and generates short-chain fatty acids (SCFAs), which can inhibit the production of proinflammatory cytokines by monocytes [23]. The loss of skin microbial diversity, together with the activation of innate immunity, can lead to chronic inflammatory conditions like acne vulgaris [24]. Thus, the interaction between the host and the skin microbiota has become an attractive target for the treatment and prevention of skin infections and autoimmune diseases. In this study, we found a significantly reduced abundance of *Cutibacterium* in the skin microbiota around the catheter, emphasizing that the skin around the exit site of the catheter is more vulnerable than normal skin, and its microbial dysbiosis warrants specific attention.

In this study, we found no significant correlation between catheter duration and alpha or beta diversity indices of the skin microbiota. This finding is unexpected, as it is generally hypothesized that longer catheter duration leads to reduced microbial diversity due to impaired skin barrier function and potential overgrowth of certain bacterial species. Such a difference might be caused by three factors: (1) The small sample size of the study might have limited the statistical reliability of detected associations between catheter duration and microbial diversity. (2) Factors other than catheter duration, such as skin condition, hygiene practices, or systemic health, might have a more substantial impact on skin microbial diversity in this patient population. (3) The distribution of catheter duration among study participants might be skewed, with an underrepresentation of patients with longer catheter durations. Thus, the impact of prolonged catheter duration on skin microbial diversity remains to be investigated in larger and longitudinal cohorts.

There are several limitations in this study. First, no culture-dependent approaches were employed to investigate the skin microbiota. The 16S rRNA gene amplicon sequencing survey is culture-independent, but it is incapable of deciphering the species and strain-level composition of the overrepresented genera. It also lacks functional validation of the disturbed strains. The direct microbiome interactive factors, such as immunoglobulin and antimicrobial peptides, are not measured in our study. Thus, the causal relationship between skin dysbiosis and skin lesions cannot be determined. Furthermore, only 10 patients were included in this study. The results are preliminary, and they call for further validation in larger cohorts. However, this study does reveal significant dysbiosis at the exit site skin of the tunneled cuffed catheter in ESRD hemodialysis patients, which has potential significance in preventing catheter-related infections.

In conclusion, there is often significant dysbiosis in the skin microbiota around the exit site of the catheter. This may represent a potential target for the prevention and management of opportunistic skin infections.

## Data Availability

The sequencing data generated for this study can be found in the National Center for Biotechnology Information (NCBI) database, with the accession code PRJNA1061650. The sequence data is publicly available.
